# Heparin/Heparan Sulfate Proteoglycans Glycomic Interactome in Angiogenesis: Biological Implications and Therapeutical Use

**DOI:** 10.3390/molecules20046342

**Published:** 2015-04-10

**Authors:** Paola Chiodelli, Antonella Bugatti, Chiara Urbinati, Marco Rusnati

**Affiliations:** Section of Experimental Oncology and Immunology, Department of Molecular and Translational Medicine, University of Brescia, Brescia 25123, Italy; E-Mails: paola.chiodelli@unibs.it (P.C.); antonella.bugatti@unibs.it (A.B.); chiara.urbinati@unibs.it (C.U.)

**Keywords:** angiogenesis, glycomic, interactome, heparin, heparan sulfate proteoglycan

## Abstract

Angiogenesis, the process of formation of new blood vessel from pre-existing ones, is involved in various intertwined pathological processes including virus infection, inflammation and oncogenesis, making it a promising target for the development of novel strategies for various interventions. To induce angiogenesis, angiogenic growth factors (AGFs) must interact with pro-angiogenic receptors to induce proliferation, protease production and migration of endothelial cells (ECs). The action of AGFs is counteracted by antiangiogenic modulators whose main mechanism of action is to bind (thus sequestering or masking) AGFs or their receptors. Many sugars, either free or associated to proteins, are involved in these interactions, thus exerting a tight regulation of the neovascularization process. Heparin and heparan sulfate proteoglycans undoubtedly play a pivotal role in this context since they bind to almost all the known AGFs, to several pro-angiogenic receptors and even to angiogenic inhibitors, originating an intricate network of interaction, the so called “angiogenesis glycomic interactome”. The decoding of the angiogenesis glycomic interactome, achievable by a systematic study of the interactions occurring among angiogenic modulators and sugars, may help to design novel antiangiogenic therapies with implications in the cure of angiogenesis-dependent diseases.

## 1. The Process of Neovascularization

Angiogenesis is the process of formation of new blood vessel from pre-existing ones. It plays key roles in embryonic development, inflammation and wound repair. Moreover, it is involved in several pathologies, among which tumor growth and metastasization [1]. In effect, the local, uncontrolled release of angiogenic growth factors (AGFs) and/or alterations of the production of natural angiogenic inhibitors, with a consequent alteration of the angiogenic balance [2], are responsible for the uncontrolled neovascularization that takes place during tumor growth [3].

**Figure 1:**
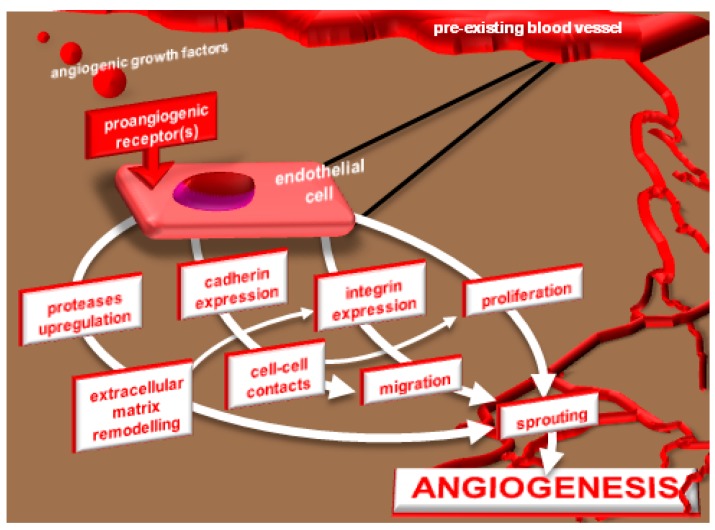
The process of tumor angiogenesis: AGFs released by tumor cells originate a chemotactic gradient that, reaching the ECs of a pre-existing vessel, stimulates an array of biological activities and phenotypical changes collectively known as “angiogenic phenotype” that lead to solid sprouts protruding from the original vessel. Then, AGFs and newly deposed ECM components orchestrate vascular morphogenesis by which sprouting ECs organize into tubes with functional lumens.

Angiogenesis is a multi-step process that leads endothelial cells (ECs) stimulated by AGFs to acquire the so called “angiogenic phenotype”. It begins with the release of effectors (*i.e.*, proteases urokinase-type plasminogen activator and matrix metalloproteinases), that degrade the extracellular matrix (ECM) creating a permissive environment for the migration and proliferation of activated EC that in this way originate solid sprouts into the stromal space, a process regulated by lateral cell-cell adhesion and ECM interactions mediated by a tightly time-regulated expression of cadherins, integrins and ECM components [4,5,6]. Lately, ECs present in the sprouts undergo “morphogenesis”, consisting in their organization in “capillary-like structures” that will mature in functional vessels, a process that still requires the proteolytic machinery [7], integrins [4] and junctional adhesion molecules [5] that, again, are controlled by the activity of AGFs and their tyrosine kinase receptors (TKRs) [8] (Figure 1).

The vascular endothelial growth factors (VEGFs) family comprises six subgroups of proteins: VEGF-A, B, C, D and E and placental growth factor (PlGF), with VEGF-A representing the most important member involved in angiogenesis. Through alternate mRNA splicing, the *VEGF-A* gene codifies for various isoforms that differ by the presence or absence of a short C-terminal heparin-binding domain. VEGFs differently interact with three distinct TKRs (VEGFRs) expressed on ECs, among which VEGFR2 (KDR) seems to be the primary pro-angiogenic receptor. It derives that the VEGF-A/VEGFR2 system represents by far the most studied target for the development of antiangiogenic drugs. However, to induce a full angiogenic response in ECs, VEGF-A also needs to interact and activate integrin α_v_β_3_ [9], neurophilin-1 (NRP-1) [10] and heparan sulfate proteoglycans (HSPGs) (discussed below).

Up to 22 members of the fibroblast growth factor (FGF) family have been identified, many of which endowed with angiogenic activity [11]. Prototypic FGF2 is a pleiotropic factor that, in addition to ECs, also acts on other cell types by interacting with a family of four TKRs named FGFRs [12]. Prototypic FGFR1 is widely expressed on ECs and its interaction with FGF2 triggers the activation of complex pro-angiogenic program [13]. However, to induce a full angiogenic response, FGF2 also needs to interact with integrin α_v_β_3_ [14], ganglioside GM1 [15], NRP-1 [16] and HSPGs (discussed below). Beside VEGFs and FGFs, many other canonical and non-canonical AGFs induce neovascularization (see Section 3.1). These AGFs can act either directly, by inducing EC to acquire the angiogenic phenotype (as typically done by VEGF-A or FGF-2) or indirectly, by inducing the production of AGFs by ECs or by other cells [as done by high mobility group box 1 (HMGB1) [17], fibronectin (FN) [18], heparanase [19] and activated blood coagulation factor X (FXa) [20]].

A tight correlation exists between angiogenesis and inflammation [1] during which macrophages produce AGFs and cytokines endowed with direct or indirect angiogenic capability [21]. Accordingly, VEGF-A synergizes with tumor necrosis factor (TNF)-α [22] and CXCL8 [23], while CCL2 synergizes with both VEGF-A [24] and FGF2 [25] in inducing angiogenesis. A tight correlation also exists between angiogenesis and viral infection [26], with some viral proteins released by infected cells that exert an angiogenic activity. In effect, during HIV infection, the transactivating factor Tat can synergize with both VEGF-A [27] and FGF2 [28] to induce neovascularization. It derives that, in a given physiopathological setting, neovascularization is almost always the result of the simultaneous actions of different AGFs, as also demonstrated in advanced stages of human tumors, characterized by a marked vascularization and the simultaneous expression of different AGFs at high levels [29,30].

These considerations had a deep impact in the field of antiangiogenic drug discovery. In effect, the possibility to inhibit neovascularization *in vivo* by using inhibitors selectively directed against a single AGF is unlikely, as sustained by the observation that the numerous antiangiogenic drugs so far developed turned out to be of little therapeutical benefit in clinical trials [31]. This failure calls for more wide-ranging studies functional to the identification of common biochemical/biological themes shared by different AGFs to be exploited in the design of multitarget drugs able to inhibit simultaneously different AGFs (see Section 5).

Natural antiangiogenic compounds are a heterogeneous group of proteins, polysaccharides and glycosphingolipids present in body fluids and ECM whose common theme is the ability to bind and sequester AGFs hampering their interaction with ECs [6]. Alternatively, some of them can act directly on ECs inducing their apoptosis or a decrease of their responsiveness to AGFs.

## 2. Heparin and HSPGs

Heparin and/or HSPGs bind to almost all the AGFs, to some pro-angiogenic receptors and even to some angiogenesis effectors, exerting modulations that can be even opposite and emerging as preferential target (or template) for the development of novel multitarget antiangiogenic drugs.

**Figure 2:**
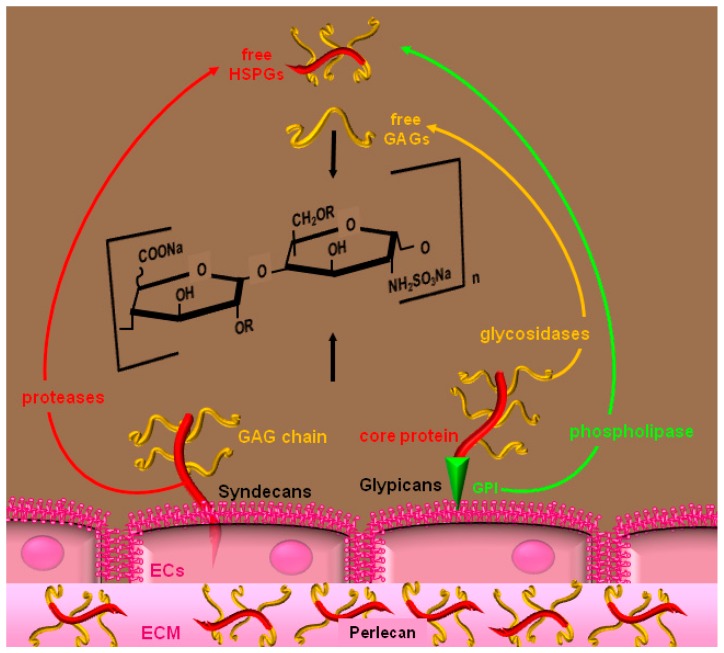
Schematic representation of the main HSPGs species syndecans, glypicans and perlecan and of their GAG chains. R = H, SO_3_Na. See text for further details.

Glycosaminoglycans (GAGs) are unbranched anionic polysaccharides (Figure 2) found as free molecules (such as heparin) or as proteoglycans composed of one or more GAG chains attached to a core protein. Heparin is synthesized by mast cells as a proteoglycan with very high molecular weight GAG chains that are then depolymerized by endoglycosidases to obtain the final product. HSPGs are instead present in almost all the cell types segregated into intracellular granules, associated with the plasma membrane or to the ECM and even in soluble forms after their mobilization [32].

The biosynthesis of heparin/heparan sulfate (HS) consists of three phases: addition of the linkage region to the core protein, chain elongation and chain modifications [33]. All the modifications are incomplete *in vivo*, so that not all the sugar residues are effectively modified. Also, since *2-O-* and *6-O*-sulfation occur only after C5 epimerization (that in turn needs the previous *N-*deacetylation/*N-*sulfation reaction) the distribution of *2-O*- and *6-O*-sulfate groups is restricted to *N-*sulfate regions. The partial modification of GAGs is the basis for their structural heterogeneity (*i.e.*, different chain length or the amount and distribution of sulfate groups).

In heparin, the modification process is more complete than in HS, so that its structure is more homogeneously composed by regular trisulfated disaccharide sequences made up of alternating, α-1,4-linked residues of IdoA2S and N,6-disulfate D-glucosamine (GlcNS6S) (Figure 2). These regular sequences are occasionally interrupted by nonsulfated uronic acids [either glucuronic (GlcA) or iduronic acid (IdoA)] and by undersulfated hexosamines (GlcNS, GlcNAc, GlcNAc6S). The less extensive modifications that occur during the biosynthesis of HS lead to GAG chains characterized by low IdoA content, low overall degree of O-sulfation and a heterogeneous distribution of the sulfate groups. Eventually, disaccharides containing GlcNAc or GlcNS may form clusters ranging from 2 to 20 adjacent GlcNAc-containing disaccharides and 2–10 adjacent GlcNS-containing disaccharides. However, about 20%–30% of the chains contains alternate GlcNAc and GlcNS disaccharides units [34].

HSPGs are found associated to the surface of almost all eukaryotic cells, including ECs, at concentrations ranging between 10^5^–10^6^ molecules/cell. HSPGs can link to the plasma membrane through a hydrophobic transmembrane domain of their core protein or through a glycosyl-phosphatidylinositol (GPI) covalently bound to the core protein [33]. Transmembrane HSPGs are the syndecans, the most represented HSPGs on ECs [33] characterized by a core protein composed of a heavily glycosilated extracellular domain, a trans-membrane domain and a cytoplasmic domain that interact with the cytoskeleton and contains sequences for tyrosine phosphorylation, enabling them to transduce a signal in the cell [33] (Figure 2). Glypicans are instead GPI-anchored HSPGs, while perlecan is the most represented HSPG in endothelial ECM [35] and can be also found tethered to integrins of the EC surface [36] (Figure 2). HSPGs also exist also in soluble form following their mobilization from the cell-surface. Transmembrane HSPGs are released after proteolytic digestion of their core protein, GPI-anchored HSPGs are instead released by endogenous phospholipase [37] (Figure 2).

The biological functions of heparin/HSPGs range from simple mechanical support functions to more articulate effects on cell proliferation and differentiation. These effects are mainly due to the ability of HSPGs to act as “receptors” for adhesion molecules, cytokines, proteases, coagulation enzymes and AGFs. HSPGs associated to the basal site of the endothelium act as receptors for basement membrane proteins, while those at the luminal surface contribute to the anticoagulative properties of the vessel surface [38] and to the internalization of lipoprotein lipase [39]. The expression of HSPGs on ECs from the microvasculature (where angiogenesis takes place) is 10–15 times higher than that on ECs from the macrovasculature [40], supporting their role in the process of neovascularization (further discussed in Section 3). As HSPGs, also heparin, secreted during inflammation, exerts a variety of effects including the regulation of coagulation (through the binding to factors such as antithrombin III and heparin cofactor II [41]) and the regulation of neovascularization (further discussed in Section 3).

The modulations imposed by heparin/HSPGs to the process of neovascularization can diverge greatly, ranging from stimulation to inhibition, mainly depending on the free, cell- or ECM-associated nature of the sugar taken in consideration: cell-associated HSPGs usually act as (co)receptors for AGF, triggering and/or enhancing the process of neovascularization. The same also holds for ECM-associated HSPGs, that act as a reservoir of AGFs and protect them from proteolytic degradation. At variance, the free forms of HSPGs, as well as heparin, sequester AGFs in the extracellular environment, hampering their interaction with ECs and thus exerting an inhibitory effect on angiogenesis [42].

## 3. Molecular Bases and Biological Sequences of the Interaction of Heparin/HSPGs with Angiogenic Modulators

As already stated, the capability of heparin/HSPGs to regulate angiogenesis relies on their binding to AGFs, pro-angiogenic receptors, antiangiogenic factors and angiogenesis effectors (Table 1). At a molecular level, the sulfate groups of GAG are almost always responsible for the interaction with heparin-binding proteins. As described above, heparin/HS structure is characterized by a high sulfation heterogeneity that generates discrete GAG sequences recognized by different proteins. The decoding of the specific GAG sequences involved in the interaction with AGFs has been eagerly pursued but only seldom achieved (as in the selected cases of the antithrombin-binding [43,44] and of the FGF2-binding [45] saccharides sequences of heparin, further discussed below). Although the development of powerful novel technologies will surely help in the better understanding of principles governing specificity of HS interactions with proteins [44,46], it is now widely accepted that protein recognition by GAGs is relatively nonselective with sharing/overlap of saccharidic sequences [47,48]. Accordingly, rather than the decoding of specific sequence, the preferential involvement of sulfate groups in the interaction of a given AGF has been established (Table 2). Originally, the search for specific GAG sequences responsible for distinct interactions was functional to the design of heparin-like drugs with selected binding capability. Today however it is widely accepted that a relatively nonselective binding would confer to heparin-like drugs a higher antiangiogenic efficiency since, as already stated, angiogenesis is almost always the outcome of the simultaneous action of different AGFs.

Sulfate groups of GAGs almost invariably bind to specific basic domains present within the amino acid sequence of the proteins (Table 3). Basic domains can consist of either linear amino acid sequences or conformational domains formed by non-contiguous basic amino acids. Multiple basic domains can sometimes be found in the same protein, as in HGF (Table 3). Within the basic domains, a tight correlation exists between their affinity for heparin and the spatial arrangement of the positive charges [49]. The sequencing of the basic domains of AGFs may help in the design and production of peptides or peptidomimetics able to specifically mask HSPGs to a given AGF.

**Figure 3:**
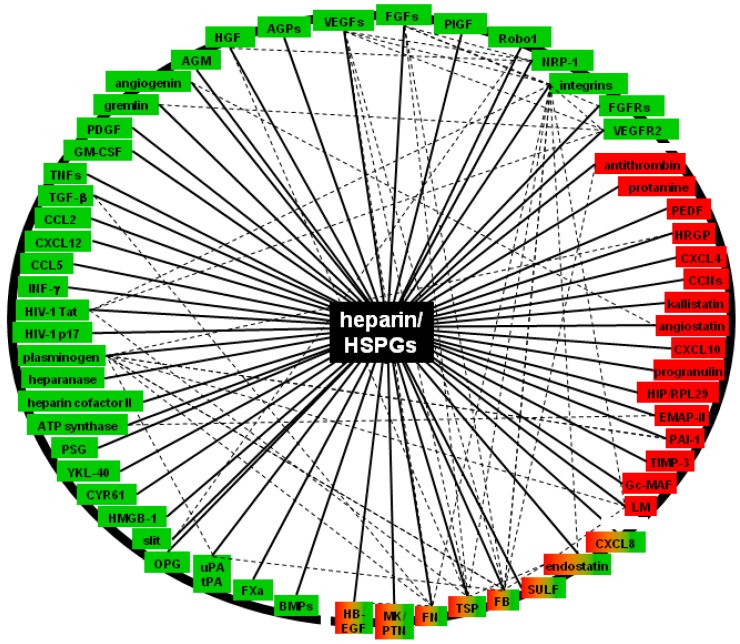
Representative angiogenesis connectivity map. Heparin/HSPGs can be ideally put at the centre of the angiogenesis connectivity map, emerging as a highly connected hub molecules. Dotted lanes indicate the mutual interaction among the various angiogenesis modulators. Green and red colours indicate a pro-and antiangiogenic effect, respectively.

The angiogenic process can be usefully represented as a “connectivity map”, in which the different modulators are tightly connected to heparin/HSPGs and among themselves (Figure 3). Connectivity maps are functional to the decoding of the “angiogenesis glycomic interactome”, whose usefulness is sustained by the works by Nunes *et al.*, that explored the changes in heparin interactome in healthy and pathological pancreas for potential biomarkers [50]. Looking to Figure 3, heparin/HSPGs emerge as highly linked “hub molecules” exploitable as target/templates for the development of therapeutics aimed at the inhibition of neovascularization in angiogenesis-dependent diseases. Here below, the structural features of the interaction of heparin/HS with the most important angiogenic modulators are reported along with the main biological consequences of such interactions.

**Table 1:** Canonical and non canonical AGFs, pro-angiogenic receptors, antiangiogenic factors and angiogenesis effectors that bind to heparin/HSPGs.

Canonical AGFs	Reference
VEGF-A	[51]
FGFs	[52]
angiopoietins	[53]
angiogenin	[54]
PlGF	[55]
platelet-derived growth factor (PDGF)	[56]
midkine/pleiotrophin	[57]
heparin-binding EGF-like growth factor (HB-EGF)	[58]
angiomodulin (AGM/TAF/mac25)	[59]
**Non Canonical AGFs and Other Regulators**	
gremlin	[60]
transforming growth factor (TGF)-β	[61]
hepatocyte growth factor (HGF)	[62]
bone morphogenetic proteins (BMPs)	[63]
interferon (IFN)-γ	[64]
TNFs	[65]
granulocyte monocyte colony stimulating factor (GM-CSF)	[66]
CXCL8	[67]
CCL2	[68]
CCL5	[69]
CXCL12	[70]
HIV-1 Tat	[71]
HIV-1 p17	[72]
pregnancy-specific β1 glycoproteins (PSGs)	[73]
α-ATP synthase	[74]
HMGB-1	[17]
CYR61	[75]
YKL-40	[76]
osteoprotegerin (OPG)	[77]
FN	[18]
fibrinogen/fibrin (FB)	[78]
heparin cofactor II	[79]
FXa	[20]
**Pro-Angiogenic Receptors**	
VEGFR2	[80]
FGFR1, 2, 3,4	[81,82,83,84,85]
neuropilin (NPR)-1	[10]
Robo	[86]
integrin α_5_β_1_	[87]
integrin α_v_β_3_	[88]
**Angiogenic Inhibitors**	**Reference**
thrombospondin-1 (TSP-1)	[89]
endostatin	[87]
CXCL4	[90]
histidine rich glycoprotein (HRGP)	[91]
protamine	[92]
CXCL10	[93]
pigment epithelium-derived factor (PEDF)	[94]
endothelial monocyte-activating polypeptide-II (EMAP II)	[74]
tissue inhibitor of metallo proteinases (TIMP)-3	[95]
laminin (LM)	[96]
serpin protease nexin-1 (PN-1)	[97]
plasminogen activator inhibitor type 1 (PAI-1)	[98]
HS-binding protein HIP/RPL29	[99]
antithrombin	[100]
**Effectors**	
sulfatase SULF-1	[101]
heparanase	[102]
tissue and urokinase-like plasminogen activators	[103]
plasminogen	[104]

### 3.1. Positive Regulators (Canonical, non-Canonical AGFs, Their Receptors and Effectors)

In this review, the term canonical AGFs refers to those AGFs that has been originally discovered for their direct, pro-angiogenic potential (*i.e.*, VEGFs and FGF2). The term non canonical AGFs refers instead to cytokines, viral proteins or other molecules that, although discovered and long studied for their roles in processes such as inflammation, coagulation or viral infections, has been then incidentally demonstrated to be endowed with the capacity to regulate also the process of neovascularization (further detailed below). Despite their different origin and roles, many of the canonical and non canonical AGFs present basic domains within their amino acid sequences (Table 3), being thus endowed with heparin-binding capacity (Table 1 and Table 2).

As mentioned above, the various VEGF-A isoforms differ by the presence or absence of a short heparin-binding domain localized into its C-terminal 55 residues (Table 3). In details, VEGF_121_ isoform lacks the heparin-binding domain and does not bind HSPGs, being found mainly as a free protein in body fluids. VEGF_189_ is instead found mainly tethered to the HSPGs of the ECM in an inactive form and its enzymatic activation generates an active form (VEGF_110_) lacking the heparin-binding domain [105]. VEGF_165_ interacts with heparin with a K_d_ equal to 11–80 nM [106,107]. It also binds to HSPGs that act as coreceptors for its subsequent interaction with VEGFR2 [108]. Although all the sulfate groups of heparin contribute to the interaction with VEGF-A, *6-O*-sulfate groups appear to be particularly important. An hexa/eptasaccharide is sufficient to bind a VEGF_165_ monomer [51].

Heparin and free HSPGs can exert opposite effects on VEGF_165_: low and high molecular weight heparins inhibit and potentiated VEGF_165_ binding to its receptors, respectively [109]. Accordingly, *in vivo*, administration of low molecular weight heparins suppress VEGF_165_-mediated angiogenesis, shortening the number and length of microvessel sprouts, while high molecular weight heparin significantly elongate microvessel [110]. Also, heparin and HS increase or inhibit VEGF_165_-binding to its receptors [111] and the consequent EC proliferation and migration [112] when administered at low and high doses, respectively.

**Table 2:** Sulfate groups of heparin/HS primarily involved in the interaction with selected angiogenic modulators.

AGF	Sulfate Groups			Reference
**VEGF-A**		*6-OSO_3_*		[51,113]
**FGF2**	*2-OSO_3_*		*NSO_3_*	[45,114]
**PlGF**	*2-OSO*	*6-OSO_3_*		[115]
**HGF**		*6-OSO_3_*		[116]
**TGF-β**			*NSO_3_*	[117]
**PDGF**	*2-OSO*	*6-OSO_3_*	*NSO_3_*	[56]
**midkine**			*NSO_3_*	[118]
**angiomodulin**	*2-OSO_3_*	*< 6-OSO_3_*	*<NSO_3_*	[119]
**HB-EGF**		*6-OSO_3_*		[120]
**gremlin**	*2-OSO_3_*	*6-OSO_3_*	*NSO_3_*	[60]
**HIV-1 Tat**	*2-OSO_3_*	*6-OSO_3_*	*NSO_3_*	[121]
**HIV-1 p17**	*2-OSO_3_*	*6-OSO_3_*	*NSO_3_*	[72]
**CXCL8**	*2-OSO*	*6-OSO_3_*	*NSO_3_*	[122]
**CXCL12**	*2-OSO_3_*		*NSO_3_*	[123]
**IFN-γ**			*NSO_3_*	[124]
**CCL2**			*6-OSO_3_*	[125]
**CCL3**	*2-OSO*	*6-OSO_3_*		[126]
**CCL21**	*2-OSO_3_*	*6-OSO_3_*		[127]
**Pro-Angiogenic Receptors**				
**FGFR1, FGFR4**		*6-OSO_3_*		[85,128]
**NRP-1**		*6-OSO_3_*		[129]
**Natural Angiogenic Inhibitors**				
**TSP-1**		*6-OSO_3_*	*NSO_3_*	[130]
**endostatin**		*6-OSO_3_*		[131,132]
**TIMP-3**	*2-OSO_3_*		*NSO_3_*	[95]
**Effectors**				
**heparanase**			*NSO_3_*	[133]
**FN**	*2-OSO*	*>>6-OSO_3_*	*>NSO_3_*	[134]

The interaction of heparin/HSPGs with FGFs occurs with K_d_ values spanning from 2 to 600 nM [135]. X-ray crystallography has identified a number of basic amino acids (Table 3) that form a “basic task” in the 3D structure of FGF2 that interact with 1–2 sulfate groups of heparin [136]. Six hexose residues of heparin are sufficient to bind 1–2 molecules of FGF2 [45]. The specific FGF2-binding sequence in HS is represented by a pentasaccharide containing the disaccharide units IdoA2S-GlcNS or IdoA2S-GlcNS6S [45], with *6-O*-sulfate groups necessary however to promote FGF2/FGFR interaction [128]. Accordingly, although a pentasaccharide is enough to bind FGF2, a decasaccharide is required to exert a modulatory effect on the AGF [137], supporting the hypothesis that heparin/HSPGs, FGF2 and FGFR1 form a ternary complex in which the GAG chain interacts with FGF2 *via 2-O*-sulfate and *N-*sulfate groups and with FGFR *via 6-O*-sulfate groups [138]. FGF2 binding to EC-surface HSPGs promotes angiogenesis *in vitro* and *in vivo* [1,139] by direct activation of intracellular signaling [140], by mediating FGF2 internalization [141], by presenting FGF2 to FGFRs in a proper conformation or by promoting the formation of the productive HSPG/FGF2/FGFR1 ternary complex [1,139]. ECM-associated HSPGs act as a reservoir for FGF2 for long-term stimulation of ECs [142]. Also, ECM degradation leads to mobilization of entrapped FGF2 with consequent activation of angiogenesis [143]. Heparin and free HSPGs bind FGF2 and protect it from heat and acidic inactivation [144] and from proteolytic degradation [145]. Also, free GAGs favor the delivery of FGF2 to the blood supply increasing its radius of diffusion [146]. At the EC-surface, heparin induces oligomerization of FGF2 [147] that is required for its full biological response [148]. Depending on its concentration, heparin can instead act as an antagonist, binding and sequestering FGF2, hampering its interaction with the ECs and inhibiting its biological activities [149].

**Table 3:** Basic domains driving the interaction of angiogenic modulators with sulfated GAGs.

AGF		Basic Domain Sequences	Reference
**VEGF-A**		R_123_R_124_R_159_	[150]
**FGF2**		K_35_R_53_K_128_R_129_K_134_K_138_K_144_K_26_N_27_R_81_K_119_R_120_T_121_Q_123_K_125_K_129_Q_134_K_135_	[151,152]
**FGF1**		N_18_K_112_K_113_N_114_	[153]
**midkine**		K_79_R_81_K_86_ K_87_R_89_K_102_	[154]
**angiomodulin**		K_89_SRKRRKGK_97_	[59]
**HGF**		K_60_K_62_R_73_R_76_ K_78_ R_512_-R-K_516_ H_645_HR-K_649_	[155,156,157]
**angiogenin**		R_31_RR_33_	[54]
**CXCL8**		H_23_K_25_K_28_K_59_R_65_K_69_K_72_	[158]
**INF-γ**		K_128_RKR_131_	[159]
**HIV-1 Tat**		R_46_KKRRQRRR_61_	[71]
**HIV-1 p17**		K_26_KKYKLKH_33_	[72]
**TGF-β_1_**		R_18_R_25_K_26_K_31_H_34_K_37_ K_60_R_94_K_97_R_107_K_110_	[117,160]
**GM-CSF**		H_15_H_83_H_87_	[161]
**HB-EGF**		K_21_RKKKGK_27_ K_31_KR_33_ R_38_KYK_41_	[162]
**CCL2**		K_5_H_66_	[163]
**Slit**		R_461_R_462_K_466_R_467_K_472_K_475_	[164]
**Pro-Angiogenic Receptors**			
**FGFR1**		K_160_K_163_K_164_H_166_K_172_H_201_K_225_	[165]
**FGFR2**		K_161_MEKRLHAVPAANTVKFR_178_	[153]
**integrin α_v_β_3_**	**α_v_subunit:**	R_65_K_446_K_489_K_520_K_535_K_645_K_646_ K_151_	[88]
	**β_3_ subunit:**		
**Angiogenic Inhibitors**			
**CXCR4**		K_77_NGR_80_ R_51_PRH_54_ K_62_ K_92_KIIKK_97_	[166]
**endostatin**		R_27_R_139_	[167]
**antithrombin**		K_115_ K_125_	[43]
**Effectors**			
**heparanase**		K_158_KFKN_162_	[168]

Angiogenin binds to heparin and EC-associated HSPGs, with X-ray crystallography suggesting that sulfate groups of heparin/HSPGs are involved in the interaction [169]. Accordingly, a basic domain has been identified in angiogenin (Table 3). The binding to heparin induces angiogenin oligomerization [54] and protect it from proteolytic degradation [170]. The binding to HSPGs causes instead angiogenin nuclear translocation of, required for its angiogenic activity [170]. Angiopoietin like-4 binds to heparin and HS with affinities ranging from 20 to 376 nM [135]. Functionally, the binding of angiopoietin-3 to EC-surface HSPGs induces retraction and loss of integrity of the EC monolayer without hampering its binding to Tie2 receptor [53]. Angiomodulin is a 30-kDa glycoprotein highly accumulated in small blood vessels of tumors that promotes capillary tube-like structures from vascular ECs [59]. Heparin and HS and heparinase treatment inhibits angiomodulin binding to cells, pointing to HSPGs as major receptors for angiomodulin-dependent pro-angiogenic activity. Midkine and pleiotrophin bind strongly to HS with a K_d_ equal to 6–16 nM [135]. In midkine, two C-terminal domains mediate the binding to heparin (Table 3), while on the GAG, all the sulfate groups contribute to the interaction, with *N*-sulfate groups resulting to be critically important (Table 2). Heparin mobilizes midkine in the blood stream from their EC-associate storage [171] and protects it from proteolytic degradation [172]. At the EC-surface, midkine binds to HSPGs with a K_d_ equal to 0.2 nM, being this interaction responsible, at least in part, for its angiogenic activity [173].

The bone morphogenetic protein antagonist Drm/gremlin exerts pro-angiogenic activity [174]. It binds heparin with a K_d_ equal to 20 nM *via* *N-*, *2-O*, and *6-O*-sulfate groups [60]. Gremlin also binds HS but not other GAGs. Accordingly, gremlin binds HSPGs of the EC surface, mediating VEGFR2 engagement and autophosphorylation, ERK_1/2_ and p38 activation, and consequent pro-angiogenic responses of ECs to gremlin [60].

HGF/c-met interaction induces angiogenesis [175] and binds to heparin and HS with K_d_ values ranging between 0.2 and 12 nM [135]. The interaction depends on basic domains present both in the *N-* and C-terminus of HGF (Table 3) that are distinct from the c-met binding domain [176], suggesting that HGF, HSPGs and c-met form a ternary complex. The HGF-binding sequence in HS contains two units of IdoA2S-GlcNS6S contiguously or alternately close to of the reducing end [177], with *6-O*-sulfate groups playing the most important role in the binding (Table 2). The minimal length of heparin that retains a HGF-binding capability is an hexa/octasaccharide, but the highest affinity is found with GAGs containing 10–12 monosaccharide units [177]. Heparin induces the release of bioactive HGF [178]. Also, heparin/HSPGs promote HGF oligomerization and HGF-dependent c-Met activation [179] increasing its mitogenic potency [179]. Immobilized heparin retains its capability to bind HGF that, in turn, retains its capability to stimulate DNA synthesis in adherent cells, suggesting that HGF can be trapped in the ECM *via* HSPG acting as a localized mitogen for adherent cells [180]. Although the data summarized above have been derived from cells other than endothelium, the possibility that HSPGs act as pro-angiogenic receptors for HGF on ECs has been inferred [155].

HIV-1 Tat is a HIV-1 encoded pro-angiogenic peptide [181] that binds heparin with a K_d_ equal to 5–64 nM [182,183]. It contains a basic domain composed by a linear stretch of positively charged amino acids (Table 3) mainly responsible for its interaction with heparin that, in turn, requires sulfation of the *2-O*-, *N-O*-, and *6-O*-positions (Table 2). An hexasaccharide is the minimal size that retains Tat-binding capability. However, the affinity of binding increases with increasing the length of the GAG, with fragments up to 18 saccharides approaching the affinity of full-size heparin [184]. Heparin can exert both agonist and antagonist effects on Tat. Indeed, it induces Tat oligomerization [184], protects it from proteolytic degradation [185] and mobilizes it from cell-associated HSPGs [185]. However, high concentrations of heparin compete with cellular receptors for Tat interaction exerting an inhibitory effect [121]. The implication of heparin/HSPGs in Tat angiogenic activity is sustained by the observation that HSPG-like low affinity, high capacity binding sites for Tat are present on ECs [186] and heparin inhibits Tat pro-angiogenic activity [187].

PSGs are the most abundant fetal proteins in the maternal bloodstream in pregnancy. PSG1 exerts a pro-angiogenic activity that depends on the presence of GAGs on ECs [73]. Accordingly, PSG1 does not bind to cells lacking surface expression of HSPGs and the binding can be restored by transfection with syndecans or glypican-1. Also, the removal of cell surface GAGs or competition with heparin completely inhibited PSG1 binding to target cells [73].

The interaction of Slit with transmembrane receptors of the Robo family provides important signals in tumor metastasis and angiogenesis. HSPGs have been demonstrated to serve as essential co-receptors in Slit signaling, by stabilizing a ternary complex in which GAG chains of HSPGs bind simultaneously with the ligand and the receptor [164]. YKL-40 is a secreted heparin-binding glycoprotein associated with a worse prognosis of various advanced human cancers. It promotes angiogenesis *in vitro* by coordinating syndecan-1 and integrin α_v_β_3_ on the EC surface thus activating FAK and ERK_1/2_ [76]. Osteoprotegerin (OPG) promotes angiogenesis *in vivo* mainly through a SDF-1/CXCR4 dependent pathway, but a role of syndecan-1 has been also inferred [77].

Apart from the canonical and non-canonical AGFs listed above, for which a correlation between heparin-binding capacity and pro-angiogenic activity has been already established, many other molecules have been studied separately for their binding to heparin/HSPGs and for their pro-angiogenic potential, so that the possible relationships between the two events represents open fields of research. The PDGFs are disulfide-bonded dimers of short or long isoforms of A and B polypeptides, with the short isoforms lacking the heparin-binding basic C-terminal domain [188]. The different PDGF isoforms are then differently entrapped in the ECM after their release by ECs [189]. Studies using desulfated heparins and heparin fragments suggest that *N*-, *2-O*-, and *6-O*-sulfate groups equally contribute to the interaction (Table 2) and that the shortest heparin fragment retaining PDGF binding capability consists of 6–8 monosaccharide units. Heparin amplifies PDGF-BB-induced PDGF-α but not -β receptor activation in HSPGs-deficient cells [190]. The involvement of TGFs in vascular function are manifold [191]. TGF-β_1_ and -β_2_, but not -β_3_ bind heparin/HSPGs [117]. In TGF-β_1_, two potential heparin-binding sites have been identified (Table 3). HS endowed with a low degree of sulfation do not potentiate TGF-β_1_ biological activity, indicating the involvement of the sulfate groups of GAGs in the interaction (Table 2). Free GAGs protect TGF-β_1_ from proteolytic degradation [192] and increase its biological activity by dissociating the growth factor from the inactivating α_2_M complex [117]. At the cell-surface, HSPGs acts as a co-receptor facilitating TGF-β_1_ interaction with its type II receptor. At variance, when naturally shed, free HSPGs sequester TGF-β_1_ inhibiting its biological activity [193]. The pro-angiogenic [194] CCL2 binds to heparin with low affinity (K_d_ = 1.55 μM) [195]. The binding occurs only with highly sulfated oligosaccharides [196], with the specific contribution of the single sulfate groups not yet assessed. An octasaccharide is the minimal heparin sequence that retains the capability to bind CCL2 inducing its dimerization [195]. CCL2 also binds to HSPGs present on EC-surface [197] and in endothelial ECM [198]. The heparin-binding domain of IFN-γ is located in its C-terminus (Table 3), while the IFN-γ binding domain in HS is composed of a domain predominantly *N*-acetylated and flanked by small *N*-sulfated oligosaccharides [159]. An hexa/octasaccharide can accommodate an IFN-γ dimer [159]. The binding to heparin protects IFN-γ from proteolytic degradation decreasing its blood clearance [199]. Heparin also prevents IFN-γ interaction with EC-surface HSPGs [200]. P17 is another HIV protein endowed with angiogenic potential [201]. An integrated approach including computational modeling, site-directed mutagenesis, chemical desulfation of heparin, and surface plasmon resonance was employed to characterize the interaction of p17 with heparin, that resulted to occur with a K_d_ equal to 190 nM [72]. The *2-O*-, *6-O*-, and *N*-*O* sulfate groups of heparin, seem to contribute equally to the binding to p17 (Table 2). Two basic motifs are present in the N and C termini of p17 and neutralization (Arg→Ala) of the former, but not of the latter, causes the loss of p17 heparin-binding capability. The N-terminal heparin-binding motif of p17 partially overlaps the CXCR1-binding domain. Accordingly, its neutralization also prevents p17 binding to the chemochine receptor. P17 binds HSPGs, but, to date, this interaction has been tentatively associated only to p17-driven cytokine up-regulation in lymphocytes [202,203]. Heparanase is an endoglycosidase that, acting on heparin and HSPGs [102] mobilizes entrapped AGFs, inducing angiogenesis with an indirect mechanism of action. However, heparanase also promotes cell adhesion, survival and signaling events independent of its enzymatic activity, suggesting that it may regulate angiogenesis by multiple mechanisms [19]. GM-CSF is a pro-angiogenic cytokine [204]. It binds heparin *via* a basic domain (Table 3) that interacts with sulfate groups of the GAGs [205]. Also TNF-α binds heparin [206] and cell-associated HSPGs [207], but the relevance of these interactions in its capability to induce angiogenesis [208] remains elusive. CYR61 is a cysteine-rich, heparin-binding protein that, once secreted, acts as an ECM-associated signaling molecule contributing to physiologic and pathologic neovascularization [75]. α-ATP synthase is a pro-angiogenic peptide that binds HS and whose activity can be inhibited by the natural antiangiogenic EMAP II [74] (further discussed below).

Beside free AGFs, heparin and HSPGs also bind some pro-angiogenic receptors associated to the surface of ECs: heparin and HSPGs bind VEGFR2 [209] and VEGFR1, stabilizing the productive ternary complex with VEGF-A at the EC-surface [210]. The binding of FGFRs with heparin/HSPGs has been the subject of a huge amount of work: FGFR1, 2, 3 and 4 bind heparin with affinities ranging between 66 nM and 3.2 μM [135]. As already reported above, *6-O*-sulfate groups of heparin mediate its binding to FGFR1 [128]. Integrins α_5_β_1_ and α_v_β_3_ bind to heparin/HSPGs through their extracellular domains [87]. Functionally, this interaction leads to the activation of FAK, Src, paxillin and ERK_1/2_ [211], but the impact of such events on angiogenesis has not been elucidated yet. NPR-1 is a multidomain receptor involved in both the development and maintenance of normal vasculature and pathological angiogenesis. It interacts with a complex network of other membrane receptors, including HSPGs, and their respective ligands. NPR-1 can be shed as a short form composed of its extracellular domain. Both membrane-associated and free forms of NRP-1 bind heparin, with possible, still unexplored, biological consequences [129]. Robo binds heparin with low affinity (Kd equal to 650 nM) [86], the consequences of such interaction have been already described above for its ligand Slit.

### 3.2. Modulators with Opposite Effects

TSP-1 is a modular, matricellular protein that regulates cell interactions with the environment. Through its different domains, TSP-1 interacts simultaneously with different cell receptors, soluble cytokines and growth factors, ECM components, and proteases. This accounts for the pleiotropic nature of TSP-1, which, depending on the relative presence of its different ligands, can induce opposite effects on angiogenesis. TSP-1 inhibits angiogenesis both indirectly (by binding and sequestering different AGFs or by masking HSPGs to their interaction) and directly (by interacting with specific EC receptors) [212]. The heparin binding domain of TSP has been located at the N-terminus of the protein and demonstrated to effectively bind syndecan-4, possibly transducing a pro-angiogenic effect rather than an inhibitory one [213,214].

Endostatin, a heparin-binding fragment of collagen XVIII, binds to heparin with low affinity (K_d_ values ranging between 1 and 25 μM [135]). It is a multifaceted molecules that exerts multiple, even opposite functions in angiogenesis depending on its monomeric or trimeric state: trimeric endostatin induces a pro-migratory phenotype in ECs that is inhibited by exogenous GAGs in a size-dependent manner, with heparin oligosaccharides containing more than 20 monosaccharide residues having optimal inhibitory activity. Monomeric endostatin inhibits instead angiogenesis induced by its trimeric counterpart or by FGF2 and VEGF-A by competing for the binding to HSPGs [215,216].

CXCL8 is a major regulator of angiogenesis during inflammation [217]. *In silico* docking of a heparin hexasaccharide to CXCL8 [158] involved a conformational basic domain (Table 3). Contrasting results have been obtained for the interaction of heparin with the monomeric or dimeric forms of CXCL8: one study demonstrated that both the forms bind to heparin with high and low affinity, respectively, with the highest affinity displayed by monomeric CXCL8 interacting with a HS octamer (K_d_ < 5 nM) [218]. Another study demonstrated instead that the affinity of monomeric CXCL8 for heparin/HS is too weak to allow binding at physiological ionic strength, whereas dimeric CXCL8 mediates binding to two sulfated domains of HS enriched with di-*O*-sulfate disaccharide unit IdoA2S-GlcNS6S- [122]. At a functional level, the binding to heparin stabilizes CXCL8, thereby prolonging its biological effect [218], and promotes its oligomerization [219]. A direct binding of CXCL8 to HSPGs on ECs has been demonstrated [220,221] but with no clear association to a pro-angiogenic effect. Rather, endothelial HSPGs are required for CXCL8 to exert its antiangiogenic activity directed toward FGF2 [222].

The heparin-binding domain of FN induces pro-angiogenic activation of ECs that is inhibited by heparin. An increase in the expression of VEGF-A is observed in FN-stimulated ECs, suggesting an indirect mechanism of action [18]. On the other hand, HSPGs serve as receptors for the first type III repeat of fibronectin that inhibits angiogenesis [223], suggesting that FN, as other modular protein can exert both pro- or antiangiogenic effects. Fibrinogen/fibrin (FB) exerts different effects on neovascularization by interacting with a wide array of AGFs, effectors, angiogenic inhibitors (*i.e.*, HRGP) and pro-angiogenic receptors [78]. FB binds heparin and HSPGs [224], inferring that these interactions may mediate at least some of its pro- or antiangiogenic effects. HB-EGF is a member of the EGF family expressed by many cell types including ECs that has been demonstrated to both induce angiogenesis [120] and to exert an antiangiogenic effect by binding to EC-surface HSPGs [225]. The extracellular sulfatases Sulf1 and Sulf2 bind to heparin with K_d_ values ranging between 0.6 and 17 nM [101]. They act by remodeling the *6-O-*sulfation state of HSPGs on the ECs surface and ameliorating the signaling of AGFs [101,226]. However, Sulf1 has been also reported to exert an antiangiogenic effect [227], calling for further studies aimed at explaining these contrasting results.

### 3.3. Natural Angiogenic Inhibitors

Protamine is a small DNA-binding cationic protein that interacts with HSPGs [228], concealing these receptors to FGF2 and FGF1 [92]. Accordingly, it inhibits FGF2-dependent angiogenesis *in vitro* [229] and *in vivo* [230]. HRGP and kallistatin are heparin-binding proteins that inhibit EC adhesion, proliferation, migration and morphogenesis induced by VEGF-A and FGF2 *in vitro* and *in vivo* [231]. In both the proteins, the heparin-binding and the antiangiogenic domains co-localize [232,233], suggesting that their antiangiogenic potential depends, at least in part, by their capability to bind HSPGs. PEDF is a collagen-binding protein abundantly distributed in various tissues that exhibits various biological functions, including the capability to inhibit angiogenesis. It directly interacts with HSPGs and its binding to collagen I is inhibited by heparin, pointing to a functional relationship between PEDF and heparin/HSPGs during angiogenesis [94]. CXCL10 exerts angiostatic activity *in vivo* and inhibits FGF2-induced EC proliferation and migration [217]. The possibility that the binding to HSPG is responsible for its antiangiogenic activity is suggested by the presence of specific HSPG binding site in ECs and by the observation that its capability to inhibit EC proliferation is abrogated by heparin [234]. Antithrombin inhibits neovascularization by blocking FGF2 and VEGF-A from forming ternary complexes with their TKRs and HSPGs. The specific heparin-binding site of antithrombin (Table 3) has been identified [44] and found to be essential for its antiangiogenic activity [43]. Also, antithrombin inhibits only the heparin-binding VEGF_165_ but not the shorter isoforms VEGF_121_ [100]. Finally, treatment of ECs with heparinase III suppress the ability of antithrombin to inhibit AGF-dependent proliferation. EMAP-II is an antiangiogenic factor containing an heparin binding motif that exerts an inhibitory effect by competing with pro-angiogenic α-ATP synthase [74]. PN-1, a protease expressed by ECs, exerts an antiangiogenic effect that does not depend on its anti-protease activity but involves its binding to HSPGs [97]. CXCL4 binds heparin with a Kd equal to 160 nM [135] and inhibits neovascularization by binding and masking HSPGs [235] and by directly binding AGFs such as FGF2 [236] and VEGF-A [237].

**Figure 4:**
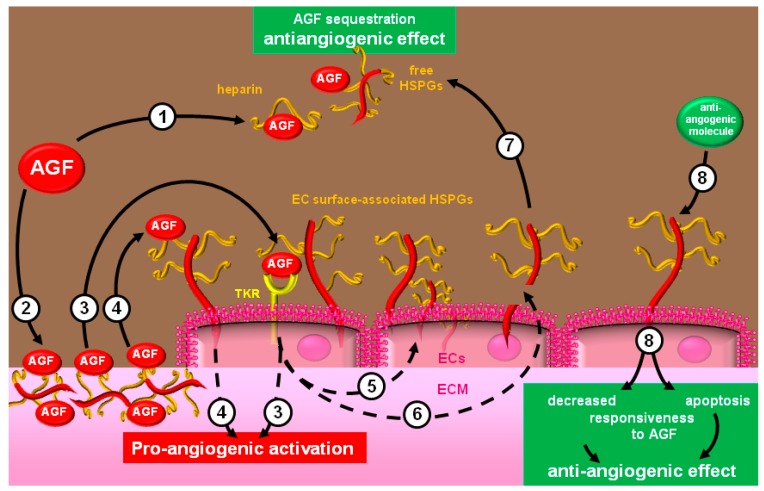
Heparin/HSPGs and the angiogenic machinery: (**1**) heparin/free HSPGs sequester AGFs hampering their interaction with ECs. (**2**) AGFs bind HSPGs of the ECM, increasing their concentration in proximity of ECs. ECM-associated AGFs are mobilized for EC long-lasting stimulation that occurs by different mechanisms: EC-associated HSPGs present AGFs to TKRs that, in turn, transduce pro-angiogenic signals in ECs (**3**). HSPGs themselves directly transduce pro-angiogenic signals following their engagement by AGFs (**4**). These same signals regulate the surface expression of HSPGs (**5**) or the production of proteases/glycosidases (**6**) that generate free HSPGs (**7**). Finally, by binding EC-associated HSPGs, antiangiogenic modulators can transduce negative signals that inhibit ECs activation (**8**).

In conclusion, an impressive number of molecules can modulate angiogenesis by binding to heparin and HSPGs. The effect exerted (stimulation or inhibition) depends on both intrinsic features of the protein (*i.e.*, modular proteins such as TSP-1, FB, FN that act as scaffold to set up multimolecular aggregates with heparin/HSPGs) and the free, cell- or ECM-associate nature of the sugar. In general, heparin/free HSPGs act as antagonists, sequestering AGFs in the extracellular environment. Conversely, EC-associated HSPGs exert a pro-angiogenic effect by acting by different mechanisms: (i) direct triggering of a signal transduction pathway in response to AGF engagement; (ii) AGFs internalization; (iii) presentation of AGFs to their TKRs in an optimal configuration. ECM-associated HSPGs act as a reservoir for AGFs that reach higher local concentration and sustain the long-term stimulation of ECs. Finally, some natural angiogenic inhibitors bind to EC-associated HSPGs inducing apoptosis or decreasing ECs responsiveness to AGFs. Interestingly, the synthesis [238] and/or mobilization [239] of HSPGs in ECs is regulated by AGFs and angiogenic inhibitors, increasing the intricacy of the system. A schematic representation of the complex interplay existing between heparin/HSPGs and the angiogenesis machinery is shown in Figure 4.

## 4. Therapeutical Exploitation of the Heparin/HSPGs Glycomic Interactome

**Figure 5:**
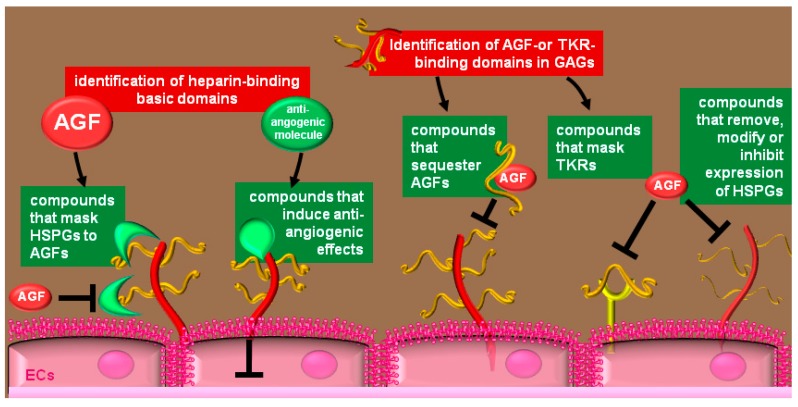
Heparin/HSPGs-related strategies to inhibit angiogenesis: from left to right: EC-surface HSPGs can be masked to AGFs by means of HSPGs-binding decoys; compounds based on natural antiangiogenic molecules can decrease EC responsiveness to AGFs or can induce EC apoptosis; heparin-like molecules can sequester AGFs, preventing their interaction with ECs or can bind and mask TKRs; some compounds decrease the expression or the sulfation degree of HSPGs at the EC surface; other can be used to directly remove HSPGs.

Due to recent improvements in glycomics [240], protein-glycan interaction have been always more taken in consideration for the design of novel therapeutic approaches. Accordingly, an equally growing number of novel glycan-based drugs entered preclinical and clinical studies [241]. The heparin/HSPGs system can be exploited to block angiogenesis in three main ways (Figure 5): (i) heparin-binding basic domains represent “templates” for the design and production of “decoy” compounds that bind and mask EC-surface HSPGs; (ii) heparin/HS represent “templates” for the design and production of drugs that, sequestering the AGFs in the extracellular environment, prevent their interaction with ECs and inhibit their pro-angiogenic effect; (iii) HSPGs can be masked to AGFs or removed, their expression can be inhibited or their composition can be modified in a form lesser recognizable by AGFs. Here below, a representative list of antiangiogenic compounds acting by the different mechanisms listed above will be reported.

### 4.1. Compounds that Bind to HSPGs

An approach for the development of antiangiogenic compounds consists in the identification of the heparin-binding domain of an AGF and the production of related synthetic peptides endowed with the capability to bind and mask HSPGs to the native AGF. The FGF2-mimicking synthetic peptide F2A4-K-NS has been produced that is able to bind and mask HSPGs to the parental AGF [242]. A synthetic peptide corresponding to exon 6a of VEGF-A binds HSPGs preventing VEGF-A interaction with the endothelial surface, EC migration and angiogenesis *in vivo* [243]. The M α5-derived peptide A5G27 binds to the GAG chains of CD44, preventing its binding to FGF2 and inhibiting angiogenesis [96]. Peptides from the heparin-binding domains of HGF (Table 3) inhibit angiogenesis independently from binding to c-met receptor [156]. The basic domain of HIV-1 Tat has been used to produce multi-valent BSA conjugates that exert antiangiogenic activity by binding not only to HSPGs but also to VEGFR2 and integrins [182]. The synthetic peptides P(65–97) from the C-terminus of pleiotrophin inhibits the angiogenic activity of pleiotrophin and FGF2 by virtue of its ability to bind heparin so to compete with the growth factors for EC-associated HSPGs [244]. LfcinB is a fragment of heparin-binding lactoferrin that inhibits angiogenesis by both FGF2 and VEGF-A by binding to HSPGs of ECs [245]. Several CXCL4-derived peptides exhibit antiangiogenic properties [246], whose mechanism of action may rely, at least in part, on their capability to bind and mask HSPGs to AGFs. It can not be ruled out that, beside masking HSPGs to AGFs, some of the compounds that bind HSPGs may also directly transduce antiangiogenic signals inside ECs. This may be the case of the CXCL4-derived peptides mentioned above [246] and of LD22-4, an 86-amino fragment of FGF2 that suppresses angiogenesis *in vivo*. Although its action is mainly mediated by NRP-1, it also contains an heparin-binding domain and requires HS for its binding to cells, inferring a possible contribution of this latter interaction to its antiangiogenic activity [247].

### 4.2. Heparin-Like Compounds that Bind AGFs

This is by far the most investigated area of antiangiogenic drug discovery. Since prototypic heparin cannot be used as a drug due to its anticoagulant activity, countless studies have been performed aimed to dissociate its antiangiogenic potential from its anticoagulant activity, leading to the development of a wide array of heparin-like candidate drugs (Table 4). VEGF-A has been considered as a main target for the development of VEGF-A-binding antiangiogenic polyanionics. Also, the possibility to block FGFs by means of heparin-like compounds raised particular interest since FGFs are pleiotropic molecules that, beside angiogenesis, directly stimulate tumor cell proliferation, a feature that can be appropriately exploited to gain efficiency in anti-FGFs based anti-cancer therapies (further discussed in Section 5). At variance with VEGF-A and FGFs, few data are available about the possibility to block the other AGFs by mean of heparin-like compounds. Again, this is in contrast with the notion that pathological angiogenesis is the outcome of the simultaneous contribution from different AGFs [29], calling for more extensive comparison of the antiangiogenic potential of heparin-like compounds on a broader array of AGFs, functional to the development of drugs endowed with multitarget activity to be employed in the treatment of angiogenesis-dependent diseases.

**Table 4:** Representative list of heparin-like compounds that bind and inhibit different AGFs.

AGF Inhibited	Heparin-Like Inhibitor	Reference
**VEGF**	chemically modified heparins	[106,110,113,248,249,250,251,252,253]
	oligosaccharides from seaweed alginic acid	[112]
	polysaccharides from *Antrodia cinnamomea*	[254]
	fucoidan	[255]
	dextran derivatives	[256]
	sucrose octasulfate	[107]
	HS mimetic compounds	[257]
	heparin-mimetic peptide SY(SO_3_)DY(SO_3_)G	[258]
	phenylacetate carboxymethyl benzylamide dextran	[209]
	phosphosulfomannan (PI-88) and derivatives	[107]
	defined GAG sequences from chondroitin sulfate	[106]
	low molecular weight fucoidan	[259]
	K5 derivatives	[260]
**FGFs**	chemically modified heparins	[37,147,248,250,261,262]
	sulfated beta-(1->4)-galacto oligosaccharides	[263]
	sulfated malto oligosaccharides	[264]
	Fucoidan	[265]
	pentosan polysulfate	[266]
	sulfated K5 derivatives	[267]
	suleparoide (HS analogue)	[268]
	β-cyclodextrin polysulfate	[269]
	Carrageenan	[270]
	HS mimetic M402	[271]
	synthetic HS	[272]
	sucrose octasulfate	[107]
	oligomannurarate sulfate JG3	[102]
	marine sulfated polymannuroguluronate	[273]
	sulfated glycoconjugates	[274]
	PI-88 and derivatives	[107]
	linked sulfated tetracyclitols	[275]
	disulfated methyl 6-azido-6-deoxy-a-dmannopyranosides	[257]
**Gremlin**	chemically modified heparins, K5 derivatives	[60]
	HS mimetic M402	[271]
	chemically modified heparins	[276]
**SDF-1**α	HS mimetic M402	[271]
	chemically modified heparins	[248]
**IL-8**	chemically modified heparins, PI-88	[107]
**HIV-1 Tat**	K5 derivatives	[277]
	pentosan polysulfate	[183]
	dextrin-2-sulphate	[278]
	sulfated polymannuroguluronate	[279]
**HIV-1 p17**	chemically modified heparins, K5 derivatives	[72]
**CXCL8**	Fucoidan	[280]
**CCL2**	Fucoidan	
	cyclodextrin sulfate	[196]
	sucrose octasulfate	
**PDGF**	heparin-derived angiogenesis inhibitor LHT7	[251]
	low molecular weight heparins	[188]
**TGF-**β**1**	Fucoidan	[281]
**IFN-γ**	HS-derived glycoconjugate mimetics	[282]
**BMPs**	HS mimetic WSS25	[63]
**heparanase**	*N-*acetylated glycol split heparin SST0001	[283]

Another relevant approach to develop heparin-like AGF “traps” devoid of anticoagulant activity is the use of non-saccharidic GAG mimetics. The prototype of this class of compounds is suramin, a polysulfonated napthylurea that inhibits different AGFs, including VEGF [284], FGF2 [285], and angiogenesis effectors such as heparanase [286]. These observations prompted various efforts to develop naphthalenesulfonate derivatives endowed with a more specific antiangiogenic profile [287,288,289,290]. Another interesting class of synthetic non-saccharidic sulfated scaffolds is represented by sulfated flavonoids [291] whose antiangiogenic potential has not been fully evaluated yet. Finally, sulfonic acid polymers are organic acids that have the strong tendency to bind tightly to proteins and have been taken in consideration as antiangiogenic antitumor compounds [292,293].

### 4.3. Heparin-Like Molecules that Bind and Mask Pro-Angiogenic Receptors

The development of these compounds is based on the observation that, beside AGFs, heparin/HSPGs can also bind pro-angiogenic TKRs with important functional consequences (see Section 3.1). In turn, this infers the possibility to design heparin-like drugs able to disrupt AGF/TKR interactions thus exerting an antiangiogenic effect: phenylacetate carboxymethyl benzylamide dextran binds and inhibits NRP-1 [209]. Modified heparins [262,294], sucrose octasulfate [107], PI-88 and its derivatives [107,295] bind and inhibit FGFR1. Chemically modified heparins bind and inhibit Robo 1 [86]. Interestingly, some heparin-like molecules can bind different receptors simultaneously, suggesting their capability to exert a multitarget antiangiogenic activity. This is the case of a marine-derived oligosaccharide sulfate that binds VEGFR2, EGF receptor and HER-2/neu [296], and of low molecular weight heparin and fucoidan that bind instead both VEGFR2 and NPR-1 [259].

### 4.4. Inhibition of EC-Surface HSPGs Expression

The proof of concept of this antiangiogenic strategy derives from the observation that antiangiogenic anti-thrombin inhibits EC proliferation by down-regulating the surface expression of perlecan [297] and that, accordingly, overexpression of perlecan antisense cDNA suppresses the autocrine and paracrine functions of FGF2 in fibroblasts [298]. Some chemical compounds have been then produced that effectively affect HSPGs expression on ECs with an impact on angiogenesis: a peracetylated 4-deoxy analogue of HS inhibits HS expression reducing also HS chain size. As a results, it prevents the binding of FGF2 and VEGF-A to ECs-associated HSPGs and inhibits angiogenesis *in vivo* [299]. Also, several fluoro-xylosides derivatives has been developed that inhibit proteoglycan synthesis in ECs preventing endothelial tube formation *in vitro* [300].

An antiangiogenic effect can be also obtained by modifying the sulfation pattern/degree of HSPGs so to decrease their binding to AGFs: sodium chlorate induces the preferential reduction of trisulfated disaccharide units of HSPGs, inhibiting VEGFR2 activation by VEGF [301] and gremlin [60] and preventing FGF2 binding and mitogenic activity [302,303]. In the same way, the GAG *6-O-*endosulfatase Qsulf1 inhibits *6-O* sulfation of heparin/HSPGs inhibiting neovascularization induced *in vivo* by FGF2 [304], while HSulf-2 inhibits bioavailability of both VEGF and FGF1 [305].

### 4.5. Removal of EC-Surface HSPGs

Depletion of EC-surface HSPGs has been so far obtained by means of the enzymes heparinases or heparitinases in controlled experimental conditions: heparinase or heparitinase treatment of ECs reduces VEGF interaction [108] and VEGFR2 phosphorylation [301]. Heparinases I and III; but not heparinase II; inhibit FGF2-dependent migration [306] and proliferation [307] of ECs *in vitro* and neovascularization *in vivo* [307]. Finally; heparinase treatment of ECs inhibits internalization of angiogenin; required of its angiogenic capability [170]. However; these results remain still without a practical translation to antiangiogenic therapy.

## 5. Conclusions

Sugars are more complex than DNA and proteins in terms of chemical structure and information density [308]. As a consequence, in respect to genomics and proteomics, glycomics suffered a delay in the development of appropriate investigative tools. This is well exemplified in the field of angiogenesis, where the proceeding of the molecular characterization of the interaction between AGFs and TKRs is far advanced in respect to that the interaction between AGFs and HSPGs. As a result, several specific inhibitors of AGFs/TKRs interaction have been developed so far that, however, turned out to be of little benefit in the treatment of angiogenesis-dependent diseases, likely because *in vivo*, neovascularization is often the result of the simultaneous actions of multiple AGFs, and the specific blockage of one AGF can be easily countervailed by the biochemical redundancy of the process.

The binding to heparin/HSPGs is a feature shared by all the known AGFs, thus representing a promising target for the development of drugs able to interfere simultaneously with multiple AGFs. Heparin and HSPGs are characterized by a high structural heterogeneity of GAG-chains that offers virtually unlimited possibilities for selective interactions with AGFs. It derives that the identification of specific GAG sequences responsible for the interaction with selected AGFs is an inherently difficult task [44]. In the meantime however, the concept that a relatively nonselective binding would confer to a heparin-like drug a higher antiangiogenic efficiency came to the limelight, prompting the search for “key” structures in GAGs that may help the design and development of multitarget heparin-like drugs. A reverse approach is represented by the production of compounds that bind and mask HSPGs to different AGFs simultaneously. The feasibility of this latter approach is sustained by the observation that synthetic peptides representing the heparin-binding domains of HGF inhibit angiogenesis induced *in vivo* not only by HGF itself but also by VEGF and FGF2 [157].

Today glycomics can benefit from a wider exploitation of bioinformatics (functional to the “*in silico*” screening of AGFs/GAGs interactions based on molecular dynamics simulation of the docking events between the binding partners [44,48,88]) flanked to technologies such as surface plasmon resonance [135], mass spectrometry (MS), matrix-assisted laser desorption/ionization MS, NMR and Raman spectroscopy, aimed to identify the conformational features required to GAGs and AGFs to bind each other [309,310].

The process of heparin-like drug discovery would involve large libraries of heparin-like compounds, making mandatory the use of oligosaccharide synthesizers [311] and carbohydrate microarrays [312]. The feasibility of this approach is sustained by “pilot” studies performed using library of heparin-derived octasaccharides [276], sulfated linked cyclitols [313], suramin-like polysulfonated distamycine derivatives [121,314], HS-mimetic glycoconjugates [282] and combinatorial library screening for heparin/HS GAGs [315]. Remarkable is the use of chemical libraries based on four component condensation reactions of isocyanides that, screened for the simultaneous inhibition of VEGF and FGF2, yield candidates drugs with interesting relationships of structure and activity [316].

Several antiangiogenic heparin-like compounds display multitarget activity: in mice, systemic administration of pentosan polysulfate inhibits the growth of tumors generated by the injection of VEGF, FGF2, pleiotrophin and midkine producing cells [30]. Pentosan polysulfate also binds and inhibits Tat [183] and FGF2 *in vitro* [317]. Accordingly, in phase I and II clinical trials it leads to stabilization of Kaposi’s sarcoma [318], a lesion in which Tat and FGF2 act synergistically [28]. Also, LHT7 could block VEGF-A, FGF2 and PDGF-B simultaneously [251]. Further interesting developments in this direction are represented by the production of an orally active low molecular weight heparin conjugate (LHTD4) that inhibit both VEGF-A and FGF2 [250].

A tight link exist among angiogenesis, virus infection, tumor and inflammation that relies on viral proteins, cytokines, receptors and adhesion molecules that “cross-contribute” to the different processes [289,290]. Interestingly, many of the heparin-binding AGFs (*i.e.*, FGFs, HGF, PDGF, CXCL8, Tat and p17) act as pleiotropic cytokines, targeting cell types other than endothelium (*i.e.*, promoting mural cell deposition, stimulating of tumor cell proliferation, maintaining an inflammatory status known to promote tumor progression or enhancing oncogenic virus replication/spreading). These proteins can be thus envisaged as “molecular overlaps” among the different pathological processes, representing promising targets for the development of multitarget drugs acting not only on angiogenesis but also on tumor growth and/or related inflammation and virus-infection.

Effectively, a simultaneous inhibition of both angiogenesis and tumor growth can be achieved by compounds that mask HSPGs to AGFs: recombinant PF-4 inhibits at once angiogenesis [319], tumor growth [235] and metastasization [320]. This same multitarget approach is at the bases of the development of an compounds generated by the fusion of a modified heparin (LHT7) with cyclic RGDyk. The resulting cRGD-LHT7 binds to both α_v_β_3_ integrin expressed on the surface of tumor and endothelial cells and to VEGF, resulting in a strong antiangiogenic, anti-tumor activity [321].

**Figure 6:**
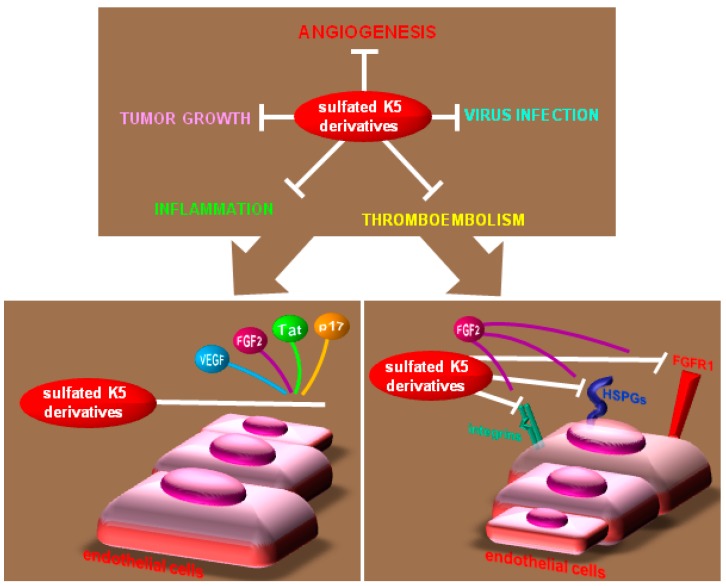
Multitarget activity of K5 derivatives. K5 derivatives interfere with different, tightly intertwined processes such as inflammation, thromboembolism, virus infection, tumor growth and angiogenesis (upper panel). Regarding angiogenesis, K5 derivatives have been demonstrated to act by binding and sequestering different AGFs (lower left panel) and to inhibit the binding of a given AGF to different pro-angiogenic receptors simultaneously (lower right panel).

Among the various antiangiogenic compounds, K5 derivatives emerge as particularly interesting “biotechnological heparin”. They are polysaccharides derived by *Escherichia coli* whose controlled sulfation confers the capability to bind (and inhibit) different AGF simultaneously, including FGF2 [267,322], VEGF [260] and the non-canonical AGFs gremlin [60], Tat [277] and p17 [72]. Also, they are able to prevent the binding of a given AGF to different pro-angiogenic receptors (*i.e.*, Tat to HSPGs and integrin α_v_β_3_ [277], FGF2 to HSPGs, FGFR1 and integrin α_v_β_3_ [267,322] (Figure 6). Finally, beside angiogenesis [42], K5 derivatives have been demonstrated to be active also in preventing virus infection [323], tumor growth [324], thromboembolism [325] and inflammation [326] (Figure 6).

Thus, K5 derivatives are representative of a “new generation” heparin-like compounds acting as global inhibitors that can disable multiple signaling networks, targeting nodal points at the crossroads of distinct molecular networks and thus expected to cause an overall failure of the disease-related system.

## Abbreviations

AGFs: angiogenic growth factors
ECs: endothelial cells
ECM: extracellular matrix
FGFs: fibroblast growth factors
FGFRs: fibroblast growth factor receptors
GAGs: glycosaminoglycans
GM-CSF: granulocyte monocyte colony stimulating factor
GPI: glycosyl-phosphatidylinositol
HB-EGF: heparin-binding epidermal growth factor
HGF: hepatocyte growth factor
HRGP: histidine-rich glycoprotein
HS: heparan sulfate
HSPGs: heparan sulfate proteoglycans
CXCL8: interleukin 8
INF-γ: interferon γ
K_d_: dissociation constant
VEGFR2: vascular endothelial growth factor receptor 2 flk-1
CCL2: monocyte chemoattractant protein 1
PDGF: platelet derived growth factor
PF-4: platelet factor 4
PlGF: placenta growth factor
Tat: HIV-1 transactivating factor
TGFs: transforming growth factors
TKRs: tyrosine kinase receptors
TNFs: tumor necrosis factors
VEGFs: vascular endothelial growth factors
VEGFRs: vascular endothelial growth factor receptors
